# Drug Repositioning of Proton Pump Inhibitors for Enhanced Efficacy and Safety of Cancer Chemotherapy

**DOI:** 10.3389/fphar.2017.00911

**Published:** 2017-12-12

**Authors:** Kenji Ikemura, Shunichi Hiramatsu, Masahiro Okuda

**Affiliations:** ^1^Department of Pharmacy, Mie University Hospital, Tsu, Japan; ^2^Department of Clinical Pharmacy and Biopharmaceutics, Mie University Graduate School of Medicine, Tsu, Japan

**Keywords:** proton pump inhibitor, drug repositioning, drug interaction, organic cation transporter 2, vacuolar H^+^-ATPase

## Abstract

Proton pump inhibitors (PPIs), H^+^/K^+^-ATPase inhibitors, are the most commonly prescribed drugs for the treatment of gastroesophageal reflux and peptic ulcer diseases; they are highly safe and tolerable. Since PPIs are frequently used in cancer patients, studies investigating interactions between PPIs and anticancer agents are of particular importance to achieving effective and safe cancer chemotherapy. Several studies have revealed that PPIs inhibit not only the H^+^/K^+^-ATPase in gastric parietal cells, but also the vacuolar H^+^-ATPase (V-ATPase) overexpressed in tumor cells, as well as the renal basolateral organic cation transporter 2 (OCT2) associated with pharmacokinetics and/or renal accumulation of various drugs, including anticancer agents. In this mini-review, we summarize the current knowledge regarding the impact of PPIs on the efficacy and safety of cancer chemotherapeutics via inhibition of targets other than the H^+^/K^+^-ATPase. Co-administration of clinical doses of PPIs protected kidney function in patients receiving cisplatin and fluorouracil, presumably by decreasing accumulation of cisplatin in the kidney via OCT2 inhibition. In addition, co-administration or pretreatment with PPIs could inhibit H^+^ transport via the V-ATPase in tumor cells, resulting in lower extracellular acidification and intracellular acidic vesicles to enhance the sensitivity of the tumor cells to the anticancer agents. In the present mini-review, we suggest that PPIs enhance the efficacy and safety of anticancer agents via off-target inhibition (e.g., of OCT2 and V-ATPase), rather than on-target inhibition of the H^+^/K^+^-ATPase. The present findings should provide important information to establish novel supportive therapy with PPIs during cancer chemotherapy.

## Introduction

Proton pump inhibitors (PPIs) inhibit gastric acid secretion by interaction with the H^+^/K^+^-ATPase in gastric parietal cells. Because PPIs have high safety and tolerability, they are the most commonly prescribed drugs for the treatment of gastroesophageal reflux disease and peptic ulcer disease (Zimmermann and Katona, 1997; Sachs et al., 2006; Shin and Kim, 2013). Interestingly, it has been estimated that approximately 20% of cancer patients are treated with PPIs to alleviate the symptoms of gastroesophageal reflux (Smelick et al., 2013). In clinical studies, many investigators have reported that co-administration of PPIs affect the development of side effect and the efficacy of anticancer agents (Suzuki et al., 2009; Santucci et al., 2010; Reeves et al., 2014; Ikemura et al., 2016, 2017). Therefore, the effect of PPIs on the efficacy and safety of anticancer agents needs to be investigated thoroughly.

Drug repositioning, the process of finding novel indications of previously approved drugs, is of growing interest to academia and industry because it reduces the time and costs associated with drug development (Ashburn and Thor, 2004). Several reports revealed that PPIs inhibited not only the H^+^/K^+^-ATPase in gastric parietal cells, but also the vacuolar H^+^-ATPase (V-ATPase) overexpressed in tumor cells (Luciani et al., 2004; De Milito and Fais, 2005; Lee et al., 2015). In addition, very recent evidence has indicated that PPIs are potent inhibitors of renal drug transporters, including human organic anion transporters (hOATs) and human organic cation transporters (hOCTs) that are associated with pharmacokinetics and/or renal accumulation of various drugs, including anticancer agents (Nies et al., 2011; Chioukh et al., 2014; Hacker et al., 2015; Ikemura et al., 2016). Therefore, co-administration of PPIs could represent novel supportive therapies during cancer chemotherapy to improve the efficacy and safety of anticancer agents via inhibition of targets other than the H^+^/K^+^-ATPase.

In this mini-review, we summarize the current knowledge regarding the impact of PPIs on the efficacy and safety of cancer chemotherapeutic drugs.

## Protective Effect of PPIs on Cisplatin-Induced Nephrotoxicity by Inhibition of OCT2

In the renal proximal tubules, membrane transport proteins expressed in the apical or basolateral membranes are responsible for the urinary secretion of diverse drugs. The structures and functions of hOATs and hOCTs encoded by *SLC22A* genes have been characterized (Sweet and Pritchard, 1999; Inui et al., 2000; Sekine et al., 2000). OAT1 (*SLC22A6*) and OAT3 (*SLC22A8*) expressed in the basolateral membrane of the proximal tubules, transport various organic anions using opposite α-ketoglutarate as a driving force (Sweet and Pritchard, 1999). In contrast, OCT1 (*SLC22A1*) and OCT2 (*SLC22A2*) were reported to be driven by inside-negative membrane potentials (Busch et al., 1996; Okuda et al., 1996), mediating basolateral uptake of diverse organic cations. Moreover, at the brush-border membranes, multidrug and toxin extrusion 1 (MATE1/*SLC47A1*) mediates the extrusion of organic cations from the cells into the tubular lumen using the transmembrane H^+^ gradient as a driving force (Yonezawa and Inui, 2011a), and is considered to be responsible for the final step of urinary excretion of cationic drugs.

Cisplatin (CDDP) is a chemotherapeutic drug widely used for the treatment of various solid tumors, including lung, ovarian, and esophageal cancers (Go and Adjei, 1999). The major side effects of CDDP include nephrotoxicity, ototoxicity, myelosuppression, and peripheral neuropathy (Arany and Safirstein, 2003). Because CDDP-induced nephrotoxicity is a dose-limiting side effect that restricts its clinical application (Pabla and Dong, 2008), the development of renal protective strategies for CDDP chemotherapy is an urgent matter that requires a solution.

**FIGURE 1:**
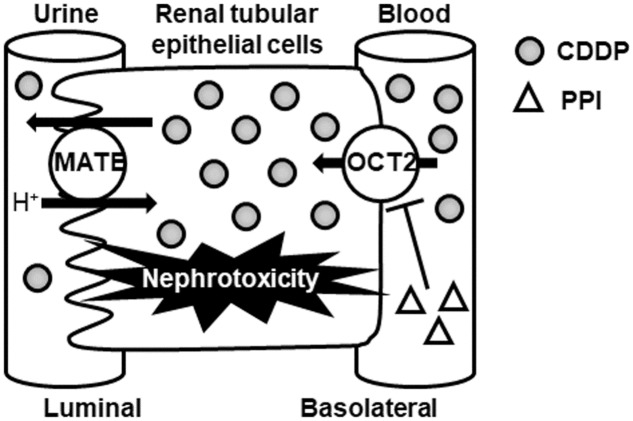
Schematic diagram of the protective effect of PPIs for CDDP-induced nephrotoxicity via OCT2. CDDP, cisplatin; MATE, multidrug and toxin excursion; OCT2, organic cation transporter 2; PPI, proton pump inhibitor.

Cisplatin is known to accumulate specifically in the kidney compared to its accumulation in other organs or in plasma (Litterst et al., 1976). As shown in **Figure 1**, OCT2 is predominantly responsible for the accumulation of CDDP in the kidney (Yonezawa and Inui, 2011b). Therefore, CDDP-induced nephrotoxicity is expected to be ameliorated by reduction or inhibition of OCT2 activity in the kidney.

Hacker et al. (2015) demonstrated that PPIs (lansoprazole and pantoprazole) inhibited OCT2-mediated transport of metformin (a typical substrate of OCT2) at a clinical dose. In addition, these PPIs were tested for clinical drug interactions with metformin in patients and healthy subjects (Ding et al., 2014; Kim et al., 2014). Therefore, co-administration of PPI may inhibit the renal accumulation of CDDP via OCT2 (**Figure 1**).

Recently, we retrospectively investigated the effect of co-administration of PPI on nephrotoxicity in 133 patients who received CDDP and fluorouracil (5-FU) therapy for the treatment of esophageal or head and neck cancer (Ikemura et al., 2017). The rate of nephrotoxicity in patients receiving PPI (12%, *n* = 33) was significantly lower than that in patients not receiving PPI (30%, *n* = 100). Severe nephrotoxicity was not observed in patients receiving PPI, whereas the rate of hematological toxicity was comparable between patients with and without PPI treatment. These findings indicate that co-administration of clinical doses of PPI ameliorates nephrotoxicity without exacerbation of hematological toxicity in patients receiving CDDP and 5-FU therapy. Although it remains unclear whether PPI directly inhibits OCT2-mediated uptake of CDDP in the kidney, co-administration of PPI during CDDP chemotherapy should be a novel approach to minimize the nephrotoxicity of CDDP using OCT2 drug interactions.

On the other hand, MATE1 is also responsible for CDDP-induced nephrotoxicity (Nakamura et al., 2010; Oda et al., 2014) as shown in **Figure 1**. Many OCT2 inhibitors also inhibit MATE1, which may increase intracellular CDDP accumulation and nephrotoxicity. Because there have been no reports regarding the effect of PPI on MATE1 activity, further study is needed to clarify the effect of PPI against MATE1-mediated transport of CDDP.

## PPIs Enhance the Antitumor Effects and Sensitivities of Anticancer Agents by Targeting V-Atpase in Tumor Cells

**FIGURE 2:**
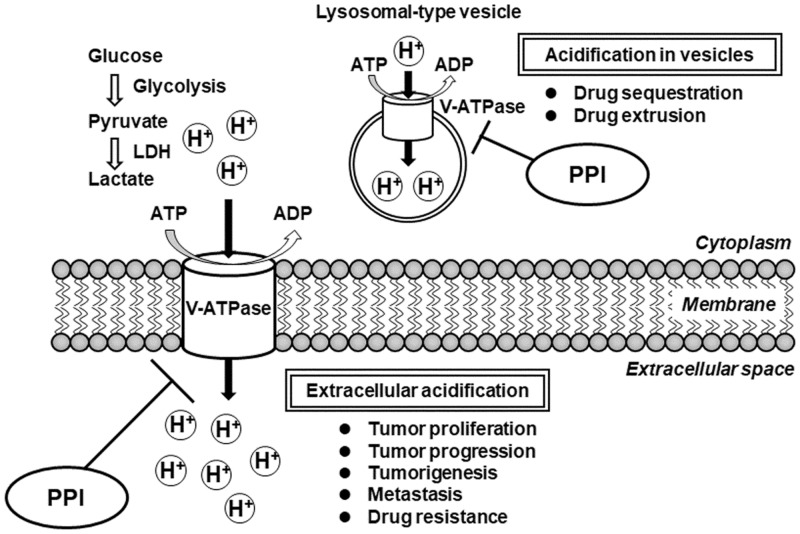
Schematic diagram of the impact of V-ATPase inhibition by PPIs for proliferation, progression, tumorigenesis, metastasis, and drug resistance in tumor cells. ADP, adenosine diphosphate; ATP, adenosine triphosphate; LDH, lactate dehydrogenase; PPI, proton pump inhibitor; V-ATPase, vacuolar H^+^-ATPase.

As shown in **Figure 2**, the V-ATPase is an ATP-dependent proton pump that transports H^+^ across both intracellular and plasma membranes to regulate intracellular and extracellular pH (Forgac, 2007). In tumor cells, increased glucose consumption via glycolysis leads to the production of lactic acid and H^+^ ions (Warburg, 1956). Because this cytoplasmic acidification is detrimental to the cells, overexpression of V-ATPase maintains an appropriate neutral cytoplasmic pH in the tumor cells, and consequently causes extracellular acidification (Nelson and Harvey, 1999). Lee et al. (2015) found that elevated expression of *V-ATPase* mRNA was significantly associated with poor survival in ovarian cancer patients. Extracellular acidification in tumor cells is known to be involved in proliferation, tumorigenesis, drug resistance, metastasis, and tumor progression (Fais et al., 2007). Inhibition of V-ATPase causes loss of the pH gradient across the plasma membranes, increasing the extracellular pH and decrease the intracellular pH, leading to slowed growth and increased cell death (De Milito et al., 2010). Furthermore, some human tumor cells exhibit elevated V-ATPase activity in intracellular lysosomal-type vesicles, leading to drug sequestration in intracellular acidic vesicles and drug extrusion from the cells through the secretory pathway (Altan et al., 1998; Raghunand et al., 1999). The acidification in intracellular vesicles is also involved in resistance to cancer chemotherapeutic drugs. Therefore, V-ATPase should be considered a promising target in the development of anticancer therapeutics.

Various prior studies have reported inhibitory effects of V-ATPase against cancer growth and metastasis in *in vivo* animal models. In mice implanted with human hepatocellular carcinoma cells, the knockdown of V-ATPase by siRNA markedly decreased primary tumor growth and suppressed intrahepatic and pulmonary metastases (Lu et al., 2005). Furthermore, the knockdown of V-ATPase by lentivirus-mediated shRNA in a 4T1 mouse model of metastatic breast cancer reduced tumor formation and decreased metastasis to the lung, liver, and bone, and consequently improved survival (Feng et al., 2013).

Interestingly, inhibition of V-ATPase could also lead to the activation of protective cellular responses (Stransky et al., 2016). Graham et al. (2014) demonstrated that bafilomycin A1, a selective V-ATPase inhibitor, upregulated mitogen-activated protein (MAP) kinases and significantly reduced tumor growth in MCF7 and MDA-MB-231 mouse xenografts. In addition, the inhibitory effect of combination treatment of bafilomycin A1 and sorafenib [an extracellular signal-regulated kinase 1/2 (ERK1/2) inhibitor] for breast tumor growth and metastasis in mice was higher than that of single administration of bafilomycin A1 or sorafenib. Thus, these findings suggest that the co-treatment of V-ATPase inhibitor with anticancer agents has synergistic antitumor effects.

Although PPIs are clinically used as H^+^/K^+^-ATPase inhibitors for the treatment of gastroesophageal reflux and peptic ulcer diseases, several *in vitro* and *in vivo* studies have addressed that PPIs also inhibit V-ATPase. Luciani et al. (2004) demonstrated that pretreatment with PPIs (omeprazole and esomeprazole) enhanced the effects of various anticancer agents (CDDP, 5-FU, and vinblastine) via inhibition of V-ATPase in cell lines derived from human melanoma, adenocarcinoma, and lymphoma. Moreover, an *in vivo* study demonstrated that oral pretreatment with omeprazole enhanced sensitivity against CDDP in mice engrafted with melanoma cells (Luciani et al., 2004). Furthermore, *in vivo* experiments were performed to confirm the synergistic effects of omeprazole and paclitaxel on tumors in orthotopic and patient-derived xenograft mouse models (Luciani et al., 2004). Interestingly, they demonstrated that PPI treatment in tumor cells increased both the extracellular pH and pH of intracellular vesicles, consistent with the inhibition of V-ATPase activity. Their evidences indicated that pretreatment with PPIs enhanced the antitumor effect through the inhibition of acidification in extracellular environment and/or in intracellular vesicles. Therefore, these findings suggest that co-administration or pretreatment with PPIs enhances the antitumor effect and sensitivity of anticancer agents by targeting V-ATPase in tumor cells.

## Conclusion

This mini-review suggests that PPIs enhance the efficacy and safety of cancer chemotherapy via off-target inhibition (i.e., of OCT2 and V-ATPase), rather than on-target inhibition (i.e., of H^+^/K^+^-ATPase). The present findings should provide important information for the establishment of novel supportive therapy during cancer chemotherapy.

## Author Contributions

KI and MO performed the literature searches. All authors contributed to the writing and final approval of the manuscript.

## Conflict of Interest Statement

The authors declare that the research was conducted in the absence of any commercial or financial relationships that could be construed as a potential conflict of interest.
